# Spontaneous Coronary Artery Dissection Unveiled: Pathophysiology, Imaging, and Evolving Management Strategies

**DOI:** 10.3390/jcdd12080286

**Published:** 2025-07-28

**Authors:** Constantin Andrei Rusali, Ioana Caterina Lupu, Lavinia Maria Rusali, Lucia Cojocaru

**Affiliations:** 1 Department of Internal Medicine, Ovidius University of Constanta, 145 Tomis Boulevard, 900591 Constanta, Romania; 2 Department of Cardiology, Constanta County Clinical and Emergency Hospital, Ovidius University of Constanta, 145 Tomis Boulevard, 900591 Constanta, Romania

**Keywords:** spontaneous coronary artery dissection (SCAD), acute coronary syndrome (ACS), intravascular imaging, fibromuscular dysplasia (FMD), non-atherosclerotic myocardial infarction

## Abstract

Spontaneous coronary artery dissection (SCAD) is an increasingly recognized, non-atherosclerotic cause of acute coronary syndrome (ACS), particularly in younger women. This comprehensive review outlines SCAD’s unique pathophysiology, which is linked to underlying arteriopathies like fibromuscular dysplasia, and highlights the critical role of advanced intravascular imaging for accurate diagnosis. A fundamental shift in management is detailed, with evidence favoring a conservative strategy for stable patients due to high rates of spontaneous vessel healing, reserving technically challenging invasive interventions for high-risk cases. Importantly, this review also addresses long-term outcomes, noting significant rates of recurrence and Major Adverse Cardiac Events (MACE), a high prevalence of persistent chest pain, and the central role of beta-blocker therapy in secondary prevention. Ultimately, SCAD requires a departure from standard ACS protocols towards a personalized approach that emphasizes accurate diagnosis, cautious initial management, and vigilant long-term follow-up.

## 1. Introduction

### 1.1. Definition

Spontaneous coronary artery dissection (SCAD) is a non-atherosclerotic, non-iatrogenic, non-traumatic dissection of the coronary arterial wall. Subsequently, an intramural hematoma is formed, compressing the true lumen and causing myocardial ischemia [1]. Sometimes, an intimal flap can also be visualized.

In practice, SCAD is defined as an acute coronary syndrome (ACS) caused by a tear in the intimal layer or a spontaneous intramural (medial) hemorrhage that occurs without preexisting atherosclerotic plaque or preceding trauma [2,3]. The earliest report dates back to 1931. A 42-year-old woman died suddenly after experiencing chest pain. The autopsy revealed a dissection of the right coronary artery. The case was reported in the British Medical Journal [1,4].

By definition, secondary (e.g., catheter-induced) dissections are excluded, leaving SCAD as a distinct clinical entity. Saw et al. proposed an angiographic classification of SCAD based on the aspect of lesions [5,6]

The table below (Table 1) summarizes these angiographic types:

### 1.2. Epidemiology

SCAD is an uncommon but increasingly recognized cause of ACS and myocardial infarction (MI). Historically considered rare (≈0.1–0.4% of all ACS) [9], modern studies suggest higher incidence (from 1% to 5%) [9,10,11,12], probably due to advancements in intra-coronary imaging. When analyzing the incidence of MI in younger or female patients, the incidence of SCAD rises to 40% [13]. For example, SCAD is now the etiology in roughly 22–43% of MI cases among women <50 years and ~25–35% of ACS in women under 50 [13,14,15,16]. Pregnancy-associated SCAD (occurring during pregnancy or ≤3 months postpartum) accounts for a sizable minority (≈5–30%) of SCAD cases, and SCAD is the cause of up to 40% of pregnancy-related MIs [3,17,18]. Despite improved recognition, SCAD is still underdiagnosed, and its true prevalence (especially in older patients and men) remains uncertain [17,18].

### 1.3. Demographics

SCAD overwhelmingly affects women, with mean age typically in the early 40s to early 50s [17,19]. For example, Saw et al. found a mean age of 51 years, with 98% of the cohort being women, in a tertiary SCAD cohort [19]. By contrast, traditional coronary disease is rare in this group. Men can also experience SCAD, but it is significantly less common, with female-to-male ratios of ~5–10:1 [13].

SCAD patients typically do not exhibit the typical atherosclerotic risk profile. Hypertension is present in some SCAD cases (often similar frequency as age-matched norms), but other factors like diabetes, hyperlipidemia, smoking, or obesity are infrequent [13]. One review emphasizes that SCAD patients have “different combinations of risk factors” than atherosclerotic MI patients, with traditional risk factors being rare aside from hypertension. Notably, young age or absence of risk factors does not exclude SCAD [13].

### 1.4. Risk Factors

Predisposing conditions and triggers for SCAD are chiefly non-traditional. Fibromuscular dysplasia (FMD)—a non-inflammatory arteriopathy of medium vessels—is the most common associated condition. SCAD cohorts report FMD prevalence from ≈25% to >70% (depending on the screening method) [13,17]. Other systemic arteriopathies or connective tissue disorders (e.g., Ehlers-Danlos type IV, Marfan syndrome) occur infrequently but are recognized as risks. SCAD is also linked to hormonal factors: up to 28–30% of SCAD cases in women < 50 occur during pregnancy or the early postpartum period, and even outside peripartum, there is a female predominance, suggesting an estrogen/progesterone role [17,18]. Moreover, many SCAD patients report intense emotional stress (more common in women) or extreme physical exertion (more common in men) immediately preceding the event [13]. Illicit stimulants (cocaine, amphetamines), hormonal therapies (oral contraceptives, IVF drugs), migraine, hypothyroidism, or inflammatory conditions have all been observed as comorbidities in small subsets [13,19,20].

In summary, SCAD is disproportionately seen in younger women without classic atherosclerotic risks. Its epidemiology features the following: incidence ~1–4% of ACS overall (up to ~30% of MI in young women); demographics mean age ~40–50, ~80–95% female; and risk factors involving vascular arteriopathies (FMD, connective-tissue), hormonal states (pregnancy/postpartum), and acute stressors [3,17,19].

## 2. Pathophysiology

SCAD arises from spontaneous hemorrhage within the coronary artery wall. Two principal mechanistic theories (“inside-out” vs. “outside-in”) have been proposed, but both result in an intramural hematoma that narrows the vessel lumen [17,19]:

Intimal Tear (Inside–Out Theory): A spontaneous tear in the intimal layer allows blood under arterial pressure to enter the media, creating a false lumen. This flap and false channel can then propagate along the vessel. Angiographically, this corresponds to Type 1 SCAD with visible dissection planes. Many cases with imaging-confirmed SCAD exhibit a clear intimal flap that feeds an intramural hematoma.

Medial Hemorrhage (Outside–In Theory): Alternatively, rupture of the vasa vasorum (microvessels in the arterial wall) may cause bleeding into the media without an initial intimal rupture. The expanding hematoma pushes inward to compress the true lumen. In this model, the intimal tear, if it occurs at all, is a secondary event. This mechanism is thought to explain many Type 2 SCAD lesions with diffuse hematoma and no obvious flap [3,17].

Evidence suggests that both mechanisms are operative in SCAD, perhaps to varying degrees in individual patients. Histopathology of SCAD cases often shows an intramural (medial) hematoma containing fresh hemorrhage and a thin or torn intimal layer, but distinguishing the initiating event in autopsy is difficult. Clinically, angiographic Type 1 lesions (flap) indicate an intimal tear, while Type 2/3 lesions may more often represent intramural hematoma without obvious flap [17].

### 2.1. Vascular Vulnerability

SCAD typically occurs in structurally abnormal arterial walls. The strong association with fibromuscular dysplasia (FMD) underscores this: FMD’s characteristic arterial wall dysplasia (intimal or medial thickening, elastic fiber fragmentation, aneurysms/tortuosity) likely renders vessels prone to dissection under stress. Indeed, angiographic features of SCAD (often in distal/mid-segments of the left anterior descending artery) mimic the “string-of-beads” or focal irregularities seen in FMD of renal/carotid arteries. In one series, >70% of non-atherosclerotic SCAD patients had coexistent FMD upon detailed screening. Other arteriopathies—such as inherited connective tissue disorders—have been reported in a few SCAD patients, suggesting a continuum of systemic vascular fragility. In sum, SCAD is considered a manifestation of an underlying arteriopathy, and precipitating factors (shear stress) operate on this vulnerable substrate [17].

### 2.2. Hormonal and Mechanical Factors

The predilection for middle-aged women and the significant occurrence of pregnancy-related cases suggest that hormonal and hemodynamic factors play a key role. Pregnancy and the postpartum period significantly change vascular biology. During pregnancy, both blood volume and cardiac output increase dramatically, while hormonal changes, particularly elevated levels of estrogen and progesterone, lead to remodeling of the blood vessel walls [17,18]. Progesterone and relaxin are believed to lower collagen content and weaken the middle layer of the arteries. Additionally, placental hormones and inflammatory mediators increase the permeability of blood vessels. During labor, there is a further increase in both intrathoracic and intra-abdominal pressures. These combined effects can make vulnerable coronary vessels more susceptible to dissection under stress [17,18].

Registry data indicate that about 28% of spontaneous coronary artery dissection (SCAD) cases in young women occur during the peripartum period. While most pregnancies following SCAD continue without complications, the peripartum stage is recognized as a high-risk time. Besides pregnancy, there is currently no conclusive evidence linking oral contraceptives or menopausal hormone therapy to SCAD; however, sudden hormonal changes—such as those occurring during postpartum recovery or due to high-dose fertility medications—should be considered in individual cases [17,18].

In addition to hormonal factors, acute stressors such as surges in catecholamines are frequently identified as triggers for spontaneous coronary artery dissection (SCAD). Activities like intense exercise, severe emotional distress, or the use of stimulants can temporarily increase the shear stress on the walls of the coronary arteries. These factors have been observed in up to one-third of SCAD events. For instance, actions like coughing, vomiting, performing the Valsalva maneuver, and other straining activities can induce dissection in women who are predisposed to this condition. Overall, SCAD typically results from a combination of a vulnerable artery—due to arteriopathy or hormonal changes—and an acute stressor, which may involve mechanical or adrenergic stress [17].

### 2.3. Inflammatory and Other Factors

The role of inflammation in SCAD remains unclear. Some early reports suggested eosinophilic infiltration around coronary dissections, leading to the “eosinophilic coronary periarteritis” theory. However, larger studies have not confirmed the existence of a specific inflammatory vasculitis associated with SCAD. Systemic inflammatory or autoimmune diseases are rarely linked to SCAD and do not seem to be causal in most instances. Additionally, circulating inflammatory markers in SCAD patients are generally not elevated beyond levels typically seen in ACS, and there is no established connection to inflammation of atherosclerotic plaques. Therefore, SCAD is not regarded as an inflammatory vascular disease on its own [17].

In summary, SCAD pathogenesis involves the mechanical disruption of the coronary wall, characterized by intimal tears and/or intramural hemorrhages, in the context of arterial vulnerability, including FMD and hormonal vascular changes. Proposed contributory factors include high estrogen/progesterone states (pregnancy), hereditary vascular fragility (FMD, genetic loci affecting vascular smooth muscle), and acute stressors (catecholamines, physical/emotional strain). Despite ongoing research, many aspects of SCAD’s etiology remain undetermined, underscoring the need for continued study in this emerging field [13,17].

## 3. Clinical Presentation

In approximately 95% of patients with SCAD, the main symptom was chest pain. Dyspnea was also a frequent symptom, being present in 50% of cases. Other symptoms include perspiration, nausea, palpitations, anxiety, and syncope. In around 8–10% of patients, the onset is sudden, represented by ventricular arrhythmias or cardiovascular death. STEMI was present in a wide range of patients with SCAD (26–87%), and non-ST segment elevation myocardial infarction (NSTEMI) was diagnosed in 13–69% of patients, according to different studies and registries. SCAD is also a rare possible cause of myocardial infarction with non-obstructive coronary arteries (MINOCA), although in most cases, it is associated with a lumen obstruction of more than 50% [20,21,22,23,24,25,26,27,28,29,30,31].

## 4. Diagnosis of SCAD

Although SCAD is responsible for less than 5% of all ACS, the incidence is significantly higher in specific population subgroups. Clinical studies showed that SCAD is responsible for 20–35% of MI in women under 50 years of age. Prompt diagnosis of SCAD is essential because the management is significantly different from that of atherosclerotic myocardial infarction. Alas, the diagnosis can be difficult because of the lack of specific biomarkers and clinical presentation similar to that of a typical acute coronary syndrome [4,32].

The golden standard for SCAD diagnosis remains invasive coronary angiography, being performed in an emergency setting in patients with acute coronary syndrome presentation. In the absence of noninvasive, viable diagnostic methods (computed tomography coronary angiography (CTCA) has insufficient resolution for the distal coronary segments and is not recommended in the acute phase). Recognizing the coronographic characteristics of SCAD remains the primary diagnostic tool for this condition. In recent years, the awareness of interventional cardiologists regarding SCAD has increased, leading to a more frequent detection of SCAD cases [4,32].

### 4.1. The Role of Invasive Coronary Angiography in SCAD Diagnosis

**Figure 1:**
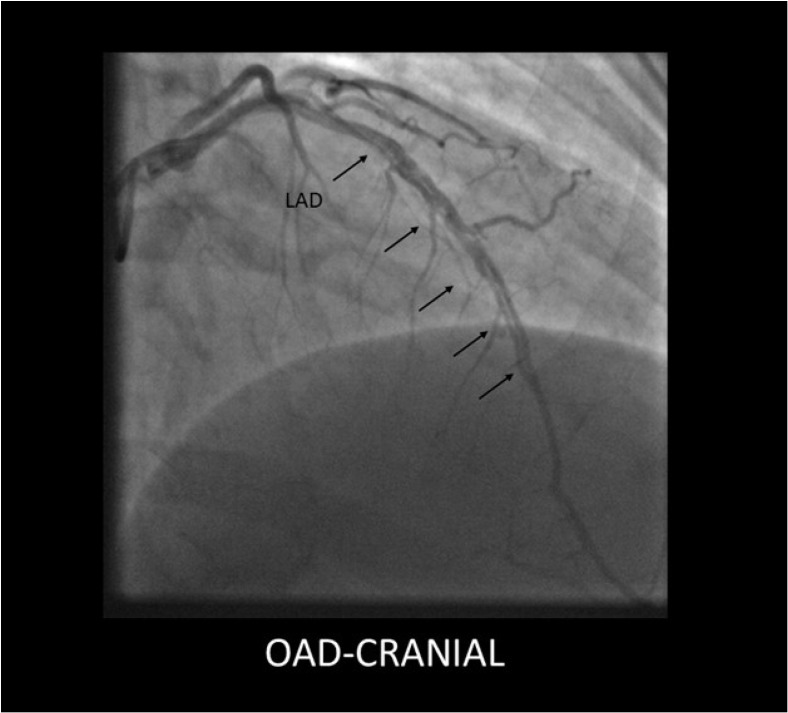
Type 1 SCAD lesion originating in the mid segment of the LAD, showing a long dissection flap (black arrows).

**Figure 2:**
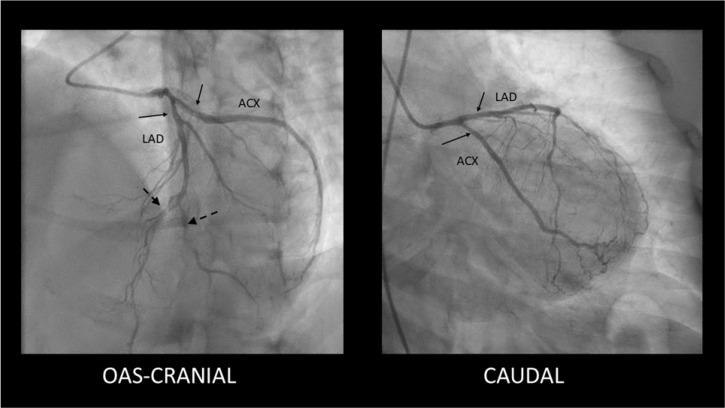
Type 2 SCAD lesions—mild, smooth stenoses located in the proximal segments of the LAD and Cx coronary arteries (solid arrows) along with distal occlusions in LAD and diagonal branch (dashed arrows).

Coronarography remains the cornerstone of SCAD diagnosis [5]. Coronographic aspects of SCAD can be distinctive, allowing for a rapid diagnosis if the interventional cardiologist has sufficient experience with this condition. The angiographic classification of SCAD is based on the aspect of lesions [5,6]:

- SCAD Type 1 (classic aspect)—double lumen and longitudinal filling defect, indicating the presence of a false lumen (wall contrast staining) and visible intimal flap. This aspect is pathognomonic for dissection and easily recognizable; however, it is present in fewer than a third of diagnosed SCAD cases. The presence of contrast inside the coronary wall (“dye hang-up” sign) suggests an already formed dissection, often with a late presentation and fewer chances of further progression [4,32] (Figure 1).

- SCAD Type 2—long, diffuse, usually tubular lesion, longer than 20–30 mm, without a visible intimal flap or double lumen. The Type 2 lesion does not ameliorate after intracoronary nitroglycerine administration (in contrast with coronary spasm). This type is the most common angiography pattern, being present in over half of SCAD cases (60–70%) [5,9,33] (Figure 2).

- SCAD Type 3—focal coronary stenosis, usually <20 mm, that mimics an obstructive atherosclerotic lesion on angiography. In reality, these lesions are also caused by an intramural hematoma (of shorter dimensions), but angiographically, they cannot be reliably differentiated from atherosclerotic plaque without additional imaging. Type 3 SCAD is identified in less than 5–10% of cases and usually requires confirmation by additional investigations—intravascular ultrasound (IVUS) or optical coherence tomography (OCT)—to identify wall dissection and exclude atheroma [5,6,33].

An angiographic “Type 4” for SCAD has recently been described, characterized by complete vessel occlusion. These total-occlusion lesions (sometimes classified as extreme Type 2) appear as acute thrombotic occlusions on angiography but are due to an occlusive intramural hematoma. In one multicenter series (the Dissezioni Spontanee Coronariche (DISCO registry), patients with Type 4 SCAD (mean age ≈ 53) presented mostly with STEMI and were often managed with PCI; Type 4 lesions were associated with LAD involvement and proximal segments. Overall, Type 4 is relatively rare and may have different acute management implications than non-occlusive SCAD [8].

The majority (approximately 85%) of patients with SCAD are initially treated conservatively based on angiographic diagnosis, without the need for confirmatory intracoronary imaging. In the Canadian CanSCAD registry, for example, only ~7.6% of patients required IVUS/OCT for definitive diagnosis, with the remainder being correctly identified only by angiogram, highlighting the need for clinicians to be familiar with SCAD angiographic patterns. Besides the aspect of the coronary lesion, other angiography clues suggest the diagnosis of SCAD instead of atherosclerotic coronary disease: high coronary tortuosity; the absence of or minimal atherosclerotic lesions in the other coronary arteries. SCAD usually affects the middle and distal coronary segments, far from the ostium, and, in contrast to atherosclerotic coronary disease, is not typically located at bifurcations. SCAD most often affects the anterior descending artery (LAD) and its diagonal branches, followed by the circumflex artery; proximal segments (including the common trunk) are rarely involved, <10% of cases. Approximately 75% of SCAD occur in the middle or distal segment of the main arteries or on secondary branches, sometimes with multiple dissections being present simultaneously (in ~10–15% of cases, SCAD lesions may be present in different arteries) [5,9,34,35,36,37,38].

An unusual diffuse stenosis in a young woman, without other atherosclerotic lesions, not influenced by nitroglycerine, should raise the suspicion of SCAD, even if an intimal flap is not seen.

Although coronary angiography is the first-line investigation and the current standard in the diagnosis of SCAD, it has numerous limitations inherent to its nature—it provides a two-dimensional image of the coronary lumen without direct information about the arterial walls. Angiography does not visualize the intramural dissection itself, but only its effect (narrowing of the lumen and possibly the intimal flap when present) [39].

Thus, in many atypical cases, dissections may go unnoticed angiographically or may be misinterpreted as atherosclerotic lesions, especially in Type 3 of SCAD (focal stenosis). Studies show that relying exclusively on the detection of contrast in the wall (the sign of Type 1 dissection) would miss a significant proportion of SCAD since the majority (over 60%) present as diffuse stenoses without a visible flap. Thus, a Type 2 or 3 SCAD may be missed, especially in the context of arterial spasm or suboptimal images [6,33,40].

Another important limitation of coronary angiography in the context of SCAD is the iatrogenic risk. The coronary arteries in patients with SCAD are more fragile and prone to extension of dissection under the action of instrumentation or contrast injections under pressure [5,9,37].

Thus, the angiographic procedure itself can, in some cases, aggravate the existing dissection or trigger a new one in the arterial ostium. The guidelines recommend that, in patients suspected of SCAD, angiography be performed with a precocious technique: avoiding deep intubation of catheters in the coronary arteries, careful monitoring of pressure curves (to detect signs of “damping” suggestive of subintimal entry), and slow and minimal injection of contrast [41].

Although there has been a theoretical concern that contrast injections may propagate the dissection, available case series have not demonstrated a significant incidence of such a complication if precautions are followed [32].

However, cases of iatrogenic extension of SCAD during angioplasty are documented. For example, guidewire manipulation may enter the false lumen, thereby extending the dissection, or balloon dilation may force the hematoma to progress proximally or distally [42].

Finally, angiography has technical limitations in visualizing very distal or small-caliber arteries, where many SCADs may occur. A dissection in a small distal branch may not be visible on angiography or may be mistaken for a spasm. In such situations, noninvasive coronary imaging may play a role. Although coronary CT angiography can reveal dissection flaps and intramural hematoma in large vessels, its resolution is insufficient for distal branches, and motion artifacts may lead to false-negative results [43].

Therefore, coronary CT is not recommended for the primary diagnosis of acute SCAD, nor its exclusion in the emergency settings [44].

However, it can be used in the evaluation of long-term evolution (for example, confirmation of healing of the dissection after a few months) or for extra coronary screening (search for dissections/aneurysms in other arteries, such as the renal, carotid arteries, in the context of the association of SCAD with FMD) [45].

Coronary angiography remains the primary method for diagnosing SCAD; however, intravascular imaging plays a crucial role in cases of diagnostic uncertainty or complex anatomy [46].

**Figure 3:**
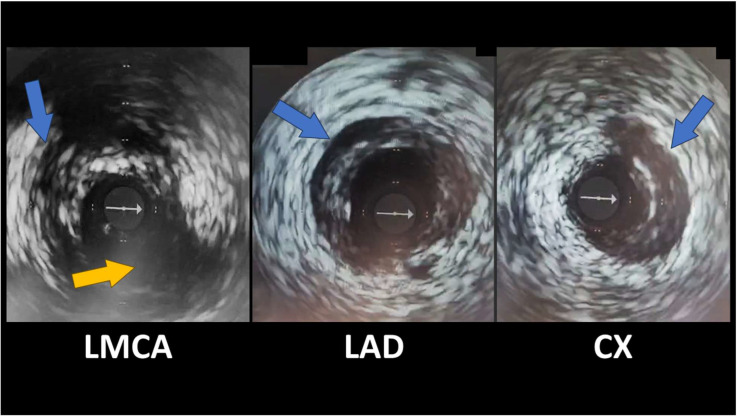
IVUS revealing spontaneous coronary artery dissections (blue arrows); Cx emergence from LMCA (yellow arrow). The white arrow indicates the IVUS guidewire.

OCT and IVUS enable direct, real-time visualization of the coronary artery wall’s structure, providing additional information compared with simple angiography (Figure 3). Both modalities can identify the characteristic features of SCAD: the presence of a double lumen (true and false) separated by an intimal flap, the delineation of an intramural hematoma, and the absence of atheromatous plaques that would suggest typical coronary artery disease [47,48].

Thus, IVUS/OCT can confirm the diagnosis in angiographically dubious lesions (e.g., Type 3 SCAD mimicking atherosclerotic stenosis) and can prevent both false negatives (missed dissections) and false positives (atherosclerotic lesions misinterpreted as dissections) [39].

In clinical practice, OCT is often preferred for visualizing coronary dissections due to its superior resolution, which enables the precise identification of the intimal flap and the extent of the hematoma within the wall [49].

However, IVUS remains a valuable method, especially in the hands of experienced operators, and can provide information in cases where OCT is not available or feasible [4,50].

A key area in which OCT/IVUS contributes is risk stratification and guiding treatment. These modalities can accurately assess the degree of compromise of the true lumen by the intramural hematoma and any points of communication between the false and true lumen [47,51].

Optical coherence tomography (OCT)—The main quality of OCT is its very high spatial resolution, ~10–20 μm axial (approx. ten times higher than IVUS), which allows detailed visualization of the arterial wall stratification (intima, media, adventitia) and identification of delicate structures, such as small intimal tears, the thin layer of residual intima between the false and true lumen, intramural thrombi, or even inflammatory infiltrates. In SCAD, OCT highlights the double-lumen morphology and the delimitation of the intramural hematoma and can localize the intimal breach (the entry point of the dissection) when it exists [47,52,53,54,55].

It also provides precise measurements of lumen diameter and dissected wall thickness, which are critical for determining stent dimensions in the event of angioplasty [56].

The significant limitations of OCT derive from its optical principle: it requires contrast injection to “wash” blood from the artery during acquisition, as infrared light does not penetrate the blood column. This necessity implies a potential risk of propagation of the dissection by the contrast jet (so-called “hydraulic injury” that can expand the hematoma) [39].

However, clinical experience suggests that if there is no prolonged stagnation of contrast in the artery, a cautiously performed OCT acquisition does not cause significant complications [19].

Another disadvantage of OCT is its limited tissue penetration (~1–3 mm in depth), which makes it difficult to assess the thickness of the entire arterial wall in large vessels or the presence of a massive hematoma [52].

Also, the window of OCT is relatively narrow longitudinally—imaging is performed in segments of a few centimeters by rapid (2–3 s) withdrawal of the catheter—and it does not allow for the continuous real-time monitoring of the segment, unlike IVUS [47].

Intravascular ultrasound (IVUS)—The main advantage of IVUS is the deep tissue penetration of ultrasound and the wide field of view, allowing assessment of the full thickness of the arterial wall (up to 4–8 mm in depth) and surrounding structures [39].

IVUS provides grayscale images of the artery, highlighting the wall architecture and being particularly useful in large vessels or dissections with marked longitudinal extension, where it can capture the proximal and distal ends of the intramural hematoma [4,57].

An important practical advantage is that IVUS does not require contrast for acquisition—the catheter with an ultrasound transducer can visualize the lumen and wall in the presence of blood, thereby eliminating the risk of contrast-induced injury and allowing for prolonged imaging, including the ability to continuously monitor the dissected segment during interventional maneuvers. IVUS has also been available for a longer time and is widely used in cardiac catheterization laboratories, with many interventional cardiologists already familiar with this technique. The limitations of IVUS are mainly related to its lower resolution (~100–150 μm) compared with OCT [47,58].

Fine details, such as a small intimal tear or a thin layer of intramural thrombus, may be overlooked with intravascular ultrasound (IVUS). Additionally, IVUS images can be challenging to interpret when different tissues have similar echogenicity. For instance, an intramural hematoma might appear as a uniform, non-echogenic area and could initially be misidentified as a lipid plaque, notably if the operator lacks experience [53,54].

The diameter of the IVUS catheter (~3F, ~1 mm) may limit access to narrow, distal, or severely tortuous coronary branches, where there is a risk of iatrogenic injury. Observational studies have shown that 70–95% of SCAD lesions heal angiographically within 1–3 months with conservative medical therapy, including aspirin and beta-blockers [15,21,55].

Intracoronary imaging reinforces this conservative strategy by allowing accurate assessment of coronary flow and residual lumen. For example, if OCT reveals that the true lumen is only moderately compressed by hematoma and there is distal Thrombolysis in Myocardial Infarction (TIMI) grade 3 flow, one can confidently opt for surveillance and conservative treatment, thereby avoiding the risks associated with an intervention. Also, IVUS/OCT can document the absence of associated intraluminal thrombosis (a frequent situation in SCAD, where the problem is wall dissection, not an occlusive thrombus), supporting the minimal use of anticoagulant and thrombolytic therapy (which are ineffective or contraindicated in SCAD). Coronary artery intervention (PCI) is only necessary in selected cases. In such cases, intravascular imaging is of great help in guiding the procedure. Angiography may overestimate the length of the dissection because irregular opacification and spasm may create the impression of a longer segment of the affected artery [59].

OCT/IVUS comes to clarify the exact limits of the dissection and the diameters of the true lumen above and below the lesion, allowing the interventional cardiologist to choose the size and length of the necessary stent correctly. This precision prevents the so-called “geographical miss”, in which the stent does not entirely cover the dissection or, conversely, unnecessarily covers a segment of the healthy artery [56].

### 4.2. Non-Invasive Imaging Methods in SCAD

#### 4.2.1. CT Coronary Angiography

Compared with invasive angiography, CT angiography does not involve catheterization. It does not carry the risk of extending an existing dissection, making it a valuable tool for evaluating SCAD in stable patients [44,60].

**Figure 4:**
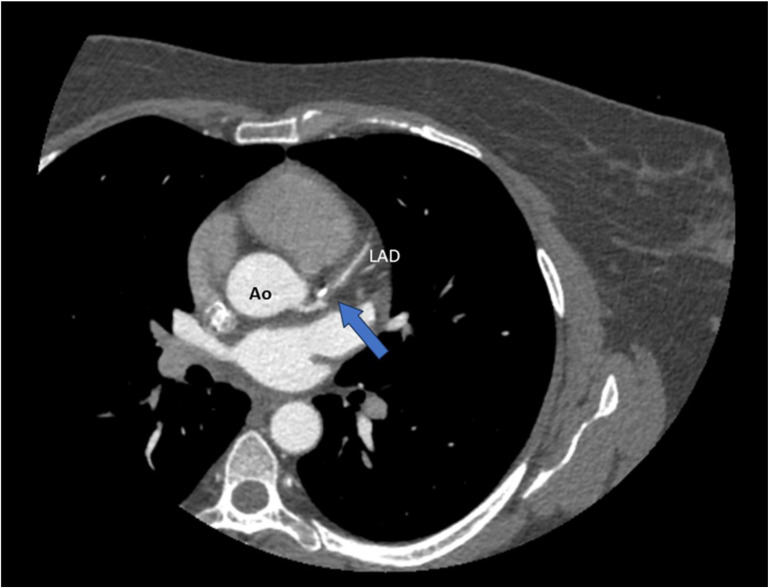
CT coronary angiography (CTCA) revealing a spontaneous dissection of the left anterior descending artery (LAD) (blue arrow). Ao = aorta.

Advantages: CT angiography can detect characteristic features of SCAD, such as tapered luminal narrowing (a gradual reduction in vessel diameter over an extended segment), abrupt narrowing, non-plaque-related occlusions, intimal flaps, and intramural hematomas manifesting as wall thickening [44] (Figure 4).

It can also indirectly reveal pericoronary inflammation (perivascular edema/fat stranding) and excessive coronary tortuosity—features frequently associated with SCAD [61,62].

An important benefit is the possibility of the examination of other arterial territories at the same time; for example, thoracoabdominal CT angiography can detect concomitant fibromuscular dysplasia (FMD) (aneurysms or “string-of-beads” appearance in the renal arteries, carotids, etc.), an associated condition in over 50% of SCAD cases [63].

CT angiography can also be used to monitor the dissected segment after a period of conservative treatment. Studies show that, by ~4–6 weeks post-event, most SCAD lesions enter spontaneous remission; for example, one report mentions complete resolution of the lesion in ~83% of cases at follow-up imaging [36].

In clinical practice, coronary CT angiography performed 1–3 months after the acute event can confirm healing of the dissection and guide the management (e.g., avoiding unnecessary interventions if the artery has spontaneously recalibrated). Despite certain advances, CT angiography has important limitations in the context of SCAD. The inferior spatial and temporal resolution of angiography can make it challenging to visualize dissections, especially in distal coronary arteries or small branches [64].

Intramural hematomas can mimic soft plaques, and specific CT criteria required for the differential diagnosis of atheroma versus dissection are not yet well defined. Thus, CT angiography may miss subtle SCAD lesions: a comparative study (with invasive angiography) showed that CT failed to detect ~20% of lesions, especially in distal segments [65].

Additionally, visualization is suboptimal for vessels with a diameter of less than 2.5 mm. Motion artifacts, particularly when the heart rate is irregular or high, can compromise image quality, often necessitating rate control or restoration of a normal sinus rhythm [66].

#### 4.2.2. Cardiac Magnetic Resonance

Through specialized sequences (Late Gadolinium Enhancement—LGE), CMR can detect and quantify myocardial infarction caused by SCAD. Typically, transmural or subendocardial gadolinium uptake is observed in the territory of the dissected artery [67]. In addition, cardiac MRI helps differentiate SCAD from other diagnoses with similar clinical presentation: myocarditis—CMR will show different gadolinium uptake (usually mesomyocardial, instead of subendocardial) and non-territorial coronary distribution; Takotsubo cardiomyopathy (“broken heart syndrome”)—CMR in Takotsubo does not reveal any late enhancement of necrosis (in the absence of infarction) [67].

## 5. Treatment

Over the past decade, as the experience on SCAD has evolved, expert consensus has shifted toward conservative management in most cases, in contrast to the aggressive revascularization algorithms applied in routine ACS. Both the American Heart Association (AHA) and the European Society of Cardiology (ESC) published scientific statements in 2018 recommending conservative management (no immediate coronary intervention) in patients with spontaneous coronary artery dissection (SCAD) who are hemodynamically stable and without ongoing ischemia, reserving revascularization for high-risk cases [36,37,68].

Observational studies have shown that dissected coronary arteries exhibit complete angiographic healing in 73–97% of patients within 4–6 weeks. In contrast, PCI in the context of SCAD has a lower success rate and an increased risk of complications compared with PCI in atherosclerotic lesions [20,42,69].

### 5.1. Invasive Treatment

Invasive treatment is reserved for cases presenting with persistent chest pain, signs of ongoing ischemia, hemodynamic instability, malignant arrhythmias, or left main coronary artery dissection [55].

A retrospective study of 189 patients showed a procedural failure rate of 53% in SCAD patients treated with PCI, compared with the expected success in atherosclerotic lesions [33].

Reasons for failure included guidewire entry into the false lumen, loss of flow after stenting, and hematoma extension, sometimes requiring emergency conversion to coronary artery bypass. In a study on 327 SCAD patients in Vancouver, only ~43% of PCIs were considered completely successful [70,71].

A study of 157 SCAD patients treated between 2005 and 2019 found that the proportion managed conservatively increased from 35% before 2013 to 89% in 2019 (*p* < 0.001). At the same time, the use of bypass surgery decreased significantly (from 23% before 2013 to 0% in 2018–2019) [68].

At a later stage (usually 4–6 weeks or 2–3 months), coronary status can be assessed by noninvasive ischemia tests or coronary CT angiography, although there is no standard protocol—some experts recommend angiographic control only if symptoms occur, otherwise assuming spontaneous healing. Studies have shown that the vast majority of patients initially managed conservatively do well, rarely requiring subsequent “rescue” interventions. In the Canadian registry of 750 SCAD patients, 86.4% were treated conservatively, and only ~2.3% of these subsequently required emergency revascularization due to clinical deterioration.

Coronary interventions (percutaneous coronary intervention (PCI) or coronary artery bypass grafting (CABG)) in the setting of SCAD are reserved for cases in which conservative therapy is unlikely to succeed or the patient is at immediate risk of significant events. Situations that require immediate revascularization include ongoing myocardial ischemia (persistent chest pain with ECG changes or electrical instability), hemodynamic instability or cardiogenic shock, dissection involving the left common trunk or main coronary ostia (which endangers a large mass of myocardium), and dissecting lesions in two proximal arteries simultaneously. Also, the presence of malignant ventricular arrhythmia at presentation may suggest persistent severe ischemia, necessitating revascularization to prevent cardiac arrest [55].

The optimal stenting strategy for SCAD is not well-established and remains an area of debate, with approaches often guided by operator experience rather than robust evidence. One common strategy aims to cover the entire dissected area, from the healthy artery segment before the dissection to beyond its distal end, to seal the false lumen. This often involves using a very long stent or multiple stents in sequence, overlapping them, and ensuring at least 5–10 mm of “safety margin” in intact arterial tissue at both ends. The technique may include the initial placement of stents at the proximal and distal extremities of the dissection (to prevent extension), followed by coverage of the dissected midsection [4].

A particular approach reported in the literature is the use of a stentless cutting balloon to fenestrate the intimal wall and allow blood from the intramural hematoma to drain into the lumen, reconstituting flow—this has been achieved in small series with a success rate (TIMI 3 flow restoration) of ~94%. However, in ~19% of patients, this technique resulted in the distal spread of the hematoma, requiring subsequent stenting [72].

Other, more minimalist, techniques are also utilized, such as stenting only the primary entry tear [73].

Furthermore, in cases where stenting is deemed necessary, bioresorbable scaffolds, such as those made from magnesium, may be considered as an alternative to permanent metallic stents [74,75].

Multiple series have shown increased complication rates: in one institution, 27.5% of SCAD patients undergoing PCI experienced acute complications during the procedure [68].

Complications may include extension of the dissection with acute coronary occlusion, requiring emergency revascularization via another route, extensive periprocedural myocardial infarction, coronary perforation or tamponade from aggressive instrumentation, and in-stent thrombosis. In one study, PCI in SCAD patients was associated with the need for emergency bypass surgery in ~13% of cases (compared with ~2% in those with conservative management) [33].

The role of CABG is exceptionally limited in the management of SCAD and should be considered only as a last resort in rare scenarios. These include left main dissection or for extensive multivessel dissections that cannot be approached percutaneously with a reasonable chance of success. CABG may also be a last resort in cases of PCI failure or persistent complete occlusion of a major artery. The procedure is also technically challenging. A primary difficulty can be distinguishing the true from the false lumen, and specific surgical principles have been developed to manage this, such as creating an open anastomosis that incorporates both lumens to reestablish flow into the true lumen [76]. In the context of SCAD, it is essential to note that native arteries can spontaneously recanalize, meaning that bypass grafts placed over these arteries may become redundant once the original vessel heals and resumes normal flow. Consequently, some surgeons prefer to use venous grafts (such as the saphenous vein) for temporary bypass rather than the internal mammary artery, accepting the possibility that the venous graft may subsequently close when native flow is restored [4].

### 5.2. Pharmacotherapy

The goals of pharmacotherapy are to prevent intraluminal thrombosis in the dissected area, improve myocardial ischemia, promote vascular healing, and reduce the risk of dissection recurrence. The therapeutic regimen should be tailored based on whether the patient underwent interventional treatment or was managed conservatively.

#### 5.2.1. Antiplatelet Agents

In SCAD, there is controversy regarding the intensity of antiplatelet therapy required. On the one hand, coronary dissection often involves intimal rupture and exposure of the subintimal layer, which triggers intraluminal thrombosis; therefore, antiplatelet agents may limit thrombus propagation and infarct extension. On the other hand, the main component of SCAD is an intramural hematoma, and theoretically, blood thinning could worsen bleeding into the arterial wall or spread the dissection. A European observational study reported that, in conservatively treated SCAD patients, the rate of major adverse cardiac events (MACE) at 1 year was significantly higher in those treated with dual antiplatelet therapy (DAPT) compared with aspirin monotherapy (18.9% vs. 6%, HR 2.62, *p* = 0.013) [57,77].

The 2023 ESC guidelines on ACS recommend the same pharmacological therapy for patients with SCAD as for those with ACS [78].

#### 5.2.2. Anticoagulants and Thrombolytics

If coronary angiography diagnoses SCAD, most experts recommend stopping systemic anticoagulation in the absence of other indications (e.g., atrial fibrillation) [37]. The reason is similar—anticoagulation may favor the expansion of the intramural hematoma and is not necessary in the absence of a large luminal thrombus. Obviously, during the PCI procedure, the use of heparin is inevitable to prevent thrombosis on catheters and stents. However, after the procedure or after diagnosis, continuous anticoagulation (e.g., heparin infusion or IIb/IIIa inhibitors) is to be avoided in SCAD. Also, long-term oral anticoagulants (Novel Oral Anticoagulants (NOAC) or warfarin) have no specific role in SCAD (unless there are independent indications, e.g., venous thromboembolism, fibrillation, etc.).

Fibrinolytic therapy is contraindicated in known SCAD. Administration of a thrombolytic (e.g., tenecteplase) in a myocardial infarction caused by SCAD may worsen the situation, as it may increase bleeding into the dissected arterial wall, leading to extension of the dissection, coronary rupture, or cardiac tamponade [36].

#### 5.2.3. Beta-Blockers

Beta-blocker therapy is one of the few pharmacological therapies with demonstrated potential benefit in SCAD. Its mechanism is clear: reduction in heart rate and contractility decreases shear stress on arterial walls, which theoretically protects the coronary arteries from the triggering forces of dissection. Clinical observations support this theory—a meta-analysis including 4206 patients with SCAD showed that beta-blocker use was associated with a significant reduction in the risk of recurrence of SCAD (relative risk ~0.51, 95% CI 0.33–0.77). Thus, beta-blockers have become first-line therapy for all patients with SCAD in the absence of contraindications [79].

#### 5.2.4. Statins

The role of lipid-lowering statin therapy after SCAD is controversial. Typically, any post-infarction patient benefits from intensive statin therapy according to guidelines due to its effects on plaque stabilization and atherosclerotic risk reduction. However, in SCAD, the non-atherosclerotic mechanism means that statins do not necessarily provide the same benefit. Furthermore, there are conflicting data on the effect of statins in this context. A retrospective study of 87 patients reported a higher incidence of recurrent SCAD in those who had received statins (50% vs. 8% at a median follow-up of ~4 years, *p* = 0.022), possibly suggesting an unfavorable effect (although it is possible that statins were preferentially prescribed to those with a higher risk profile). On the other hand, a systematic review of 295 patients found no significant association between statin therapy and the risk of recurrent SCAD. The expert consensus is that statins are not routinely indicated after a SCAD event unless there is a clear independent indication [20,79].

#### 5.2.5. Angiotensin-Converting Enzyme Inhibitors (ACEIs) and Angiotensin II Receptor Blockers (ARBs)

These classes of drugs are primarily aimed at protecting ventricular function and controlling blood pressure. In patients with SCAD who have developed left ventricular systolic dysfunction (ejection fraction (EF) < 50% post-infarction), the indications are similar to those for typical post-MI patients—an ACE inhibitor (or AT1 receptor blocker) is recommended for remodeling and prevention of heart failure, according to standard guidelines [78].

#### 5.2.6. Other Symptomatic Therapies

Many patients who have suffered SCAD continue to complain of chest pain or intermittent anginal sensations, even after objective healing of the dissection. This phenomenon of post-SCAD “chest pain syndrome” is reported in over half of patients [55].

The etiology of persistent pain can be varied, including microvascular ischemia (dysmetabolism in the coronary microcirculation induced by the dissecting event). Microvascular disorders (low coronary reserves) were objectively detected in specialized tests in more than 70% of patients with chronic post-SCAD pain [80].

There may also be non-ischemic causes, such as coronary spasms or neurogenic mechanisms. For the management of these symptoms, classic antianginals can be used. Organic nitrates (e.g., nitroglycerin as needed or isosorbide mononitrate) and calcium blockers (diltiazem, amlodipine) can be helpful, especially if vasospasm or microvascular dysfunction is suspected [4].

## 6. SCAD in Pregnancy and Postpartum

Pregnancy-related SCAD (P-SCAD) usually occurs in the third trimester of pregnancy or in the first weeks postpartum, when hemodynamic and hormonal changes are at their peak. Although P-SCAD represents a smaller percentage of total SCAD (estimated 5–10% of cases), its evolution tends to be more severe compared with non-pregnancy SCAD. The immediate prognosis can be severe—in-hospital mortality of P-SCAD is reported to be ~4%, significantly higher than other SCADs. In the acute phase, the priorities are to stabilize the mother and, secondarily, the fetus [81,82,83,84].

If SCAD occurs during pregnancy and revascularization is needed, this takes precedence—PCI can also be performed during pregnancy with radiation protection measures for the fetus and, in critical situations (e.g., left main dissection with cardiogenic shock), emergency delivery (cesarean section) followed immediately by CABG if the gestational age allows fetal viability.

Pharmacological therapy in pregnancy/postpartum has some particularities: Aspirin is safe in pregnancy (in low doses). Aspirin is frequently used for the prevention of preeclampsia anyway, so its safety profile is well established. Beta-blockers are mostly considered safe; labetalol is preferred as a first-line antihypertensive in pregnancy (it has been widely used in gestational hypertension), followed by metoprolol as an acceptable option. However, atenolol is to be avoided, being associated with low fetal weight and neonatal bradycardia [85].

P2Y12 inhibitors (e.g., clopidogrel, ticagrelor) cross the placenta and, although clopidogrel is rated FDA category B (no evidence of risk in humans, but limited data) and ticagrelor C (possible risk), their use in pregnancy is accepted only if the benefit outweighs the risk. Suppose a pregnant patient has required coronary stenting. In that case, DAPT will be maintained for at least 1 month (preferably with clopidogrel as the agent, as it has been studied more extensively in pregnancy than ticagrelor). These cases must be managed in a multidisciplinary manner. Statins are contraindicated in pregnancy (category X—teratogenic). Also, ACE inhibitors and ARBs are contraindicated (especially in the II-III trimesters, due to the risk of oligoamnios and renal failure in the fetus), replacing them with other safe antihypertensives if blood pressure control is necessary. Heparin can be used if the condition requires it (unfractionated or fractionated heparin does not cross the placenta); however, as mentioned, after diagnosing SCAD, it is preferable to discontinue anticoagulation [4].

## 7. Recurrence and Long-Term Outcomes

Long-term follow-up after SCAD reveals notable risks, even if the acute prognosis is favorable. The rate of recurrent SCAD is approximately 10–15% at 3–5 years, and these recurrences are the primary driver of Major Adverse Cardiac Events (MACE), which occur in up to 20% of patients over a similar period. Consequently, secondary prevention is crucial. The cornerstone of this strategy is long-term beta-blocker therapy, which has been shown to reduce the risk of recurrence by approximately 50%. Beyond recurrence, a common and challenging sequela is persistent chest pain syndrome, affecting over half of survivors and often managed with anti-anginal medications like nitrates or calcium channel blockers [4,5,22,86,87].

## 8. Further Research

Future research priorities should include the following:

1. Identification of Genetic Markers: Discovering genetic markers and connective tissue signatures can help better define at-risk populations, enabling preemptive counseling and screening.
2. Longitudinal Studies on Hormonal Influences: Conducting long-term studies on hormonal influences, particularly in peripartum SCAD, will help clarify the roles of estrogen, progesterone, and relaxin in arterial wall fragility.
3. Development of Standardized Imaging Protocols: Creating standardized imaging protocols can optimize early diagnosis in emergency settings while minimizing reliance on invasive techniques.
4. Evaluation of Tailored Pharmacologic Regimens: Assessing customized medication regimens is crucial, particularly regarding the risks and benefits of antiplatelet drugs and statins in patients undergoing conservative management.
5. Exploration of Quality-of-Life Outcomes: Investigating quality-of-life outcomes and the psychosocial burdens faced by SCAD survivors is crucial, with a focus on issues such as persistent angina, anxiety, and fears of recurrence.
6. Clinical Trials for Novel Therapeutics: Initiating clinical trials for innovative therapeutic strategies, including vascular-stabilizing agents or hormone-modulating therapies, is vital, particularly for selected high-risk groups.

## 9. Conclusions

Spontaneous coronary artery dissection (SCAD) is a distinct and increasingly recognized cause of acute coronary syndrome that is fundamentally different from atherosclerotic disease in its pathophysiology, patient demographic, and management. As the clinical manifestation of a broader systemic arteriopathy, its diagnosis requires a high index of suspicion, often clarified by advanced intravascular imaging like OCT and IVUS. This distinction is critical because the cornerstone of management has shifted from routine intervention towards a conservative approach for stable patients, a strategy that respects the natural history of vessel healing. Ultimately, the growing awareness of SCAD compels clinicians to move beyond a one-size-fits-all ACS protocol and adopt a more personalized approach that balances caution with vigilance for this unique condition.

## Figures and Tables

**Table 1:** Angiographic classification of SCAD.

SCAD Type (Angiographic)	Features	Comments
Type 1 (Classic)	Multiple lumens or contrast staining (visible flap) through vessel wall [7].	Pathognomonic (“flap”). <30% of SCAD; suggests an intimal tear. Often managed conservatively.
Type 2 (Diffuse)	Long, smooth narrowing (>20 mm) of the mid-to-distal artery; lumen caliber tapers over the segment [7].	The most common type. Variant 2A: normal distal segment; 2B: extends to vessel tip. Typically heals with conservative treatment.
Type 3 (Focal)	Short (<20 mm) tubular stenosis mimicking an atherosclerotic plaque [7].	Rarest. Requires OCT ^1^/IVUS ^2^ for confirmation of intramural hematoma.
Type 4 (Occlusion)	Total occlusion of the vessel, with abrupt cutoff on angiography [8].	Less common. Often presents as STEMI ^3^; may require PCI ^4^.

^1^ OCT = Optical Coherence Tomography; ^2^ IVUS = Intravascular Ultrasound; ^3^ STEMI = ST-segment elevation myocardial infarction; ^4^ PCI = Percutaneous Coronary Intervention

## Data Availability

The data underlying this article will be shared on reasonable request to the corresponding author.

## Abbreviations

The following abbreviations are used in this manuscript:

ACEI	Angiotensin-Converting Enzyme Inhibitor
ACS	Acute Coronary Syndrome
AHA	American Heart Association
ARB	Angiotensin II Receptor Blocker
CABG	Coronary Artery Bypass Grafting
CMR	Cardiac Magnetic Resonance
CT	Computer Tomography
DAPT	Dual Antiplatelet Therapy
ECS	European Society of Cardiology
FMD	Fibromuscular Dysplasia
IVUS	Intravascular Ultrasound
LAD	Left Anterior Descending (artery)
LGE	Late Gadolinium Enhancement
MACE	Major Adverse Cardiac Events
MI	Myocardial Infarction
MINOCA	Myocardial Infarction with Non-Obstructive Coronary Arteries
NOAC	Non-Vitamin K Antagonist Oral Anticoagulant
NSTEMI	Non-ST-Elevation Myocardial Infarction
OCT	Optical Coherence Tomography
PCI	Percutaneous Coronary Intervention
SCAD	Spontaneous Coronary Artery Dissection
STEMI	ST-Elevation Myocardial Infarction
TIMI	Thrombolysis In Myocardial Infarction (flow grade)
